# Monte-Carlo dosimetry and real-time imaging of targeted irradiation consequences in 2-cell stage *Caenorhabditis elegans* embryo

**DOI:** 10.1038/s41598-019-47122-7

**Published:** 2019-07-22

**Authors:** Eva Torfeh, Marina Simon, Giovanna Muggiolu, Guillaume Devès, François Vianna, Stéphane Bourret, Sébastien Incerti, Philippe Barberet, Hervé Seznec

**Affiliations:** 1 Université de Bordeaux, Centre d’Etudes Nucléaires Bordeaux Gradignan (CENBG), Chemin du Solarium, 33175 Gradignan, France; 2 CNRS, UMR5797, Centre d’Etudes Nucléaires Bordeaux Gradignan (CENBG), Chemin du Solarium, 33175 Gradignan, France; 3 Present Address: François Vianna: Institut de Radioprotection et de Sûreté Nucléaire, Bat.159, BP3, 13115 St-Paul-Lez-Durance, Cedex France

**Keywords:** Biophysics, Biological physics, Experimental nuclear physics

## Abstract

Charged-particle microbeams (CPMs) provide a unique opportunity to investigate the effects of ionizing radiation on living biological specimens with a precise control of the delivered dose*, i.e*. the number of particles per cell. We describe a methodology to manipulate and micro-irradiate early stage *C. elegans* embryos at a specific phase of the cell division and with a controlled dose using a CPM. To validate this approach, we observe the radiation-induced damage, such as reduced cell mobility, incomplete cell division and the appearance of chromatin bridges during embryo development, in different strains expressing GFP-tagged proteins *in situ* after irradiation. In addition, as the dosimetry of such experiments cannot be extrapolated from random irradiations of cell populations, realistic three-dimensional models of 2 cell-stage embryo were imported into the Geant4 Monte-Carlo simulation toolkit. Using this method, we investigate the energy deposit in various chromatin condensation states during the cell division phases. The experimental approach coupled to Monte-Carlo simulations provides a way to selectively irradiate a single cell in a rapidly dividing multicellular model with a reproducible dose. This method opens the way to dose-effect investigations following targeted irradiation.

## Introduction

Charged-particle microbeams (CPMs) present unique features for radiation biology studies: they allow the targeting of sub-cellular compartments with micrometre accuracy, to set a definite irradiation timing and a precise dose control at the single-cell scale^1–3^. In the last decades, CPMs have been extensively used to study several biological endpoints in single cells such as DNA damage and repair mechanisms, cell communication (bystander effect), low-dose effects, and radiation sensitivity of cell compartments (nucleus, cytoplasm, and mitochondria)^4–6^.

Although data from *in vitro/in cellulo* experiments on monolayer cell culture are very useful to dissect the molecular mechanisms induced by the exposure to ionizing radiation (IR), they are difficult to extrapolate to the *in vivo* response. For this reason, there is a growing interest in applying CPM irradiation techniques to *ex vivo* and *in vivo* biological models. CPMs have been gradually extended to the irradiation of three-dimensional tissue models and small multicellular *in vivo* systems to overcome these limitations^6–10^. Well-characterized biological models, such as the zebrafish *Danio rerio* embryo^11,12^, the *Bombyx mori* silkworm^13^, and the nematode *Caenorhabditis elegans* (*C. elegans*)^14,15^, are also compatible with the limited range of the particles used by CPMs. Among these models, *C. elegans* presents numerous advantages for *in vivo* investigation of radiation effects: simple culture conditions and maintenance, a rapid life cycle, a transparent body, an adult organism with only 959 somatic cells and invariant cell lineage between different animals. Moreover, *C. elegans* provides a highly characterized whole organism model where changes in genes, cells and tissues, during development is precisely defined. A wide variety of fully characterized mutants and transgenic strains is available at the *C. elegans* Genome Center (CGC).

Several studies of radiation-induced responses have been carried out using X and γ-Rays irradiation of *C. elegans*. Deng *et al*. have established a relation between the ceramide biogenesis pathway and the radiation-induced apoptosis in the germ line of *C. elegans*^16^. Using X-rays, Sendoel *et al*. have found that HIF-1 can regulate p53-mediated apoptotic cell death in response to radiation-induced damage through the secretion of a neuronal tyrosinase^17^. *C. elegans* has also been used as a biological model for space flight research, including testing the biological effects of cosmic radiation^18^.

From a practical point of view, *C. elegans* is small enough to be compatible with microbeam irradiation since the adult body is 50 µm in diameter and 1 mm in length. Its transparent body allowing a direct visualization of specific tissues makes it a unique model for studying the production and transfer of intra- and inter-tissue damage signalling in the whole organism. A few studies have described the use of *C. elegans* at CPM end-stations^14,15,19^. Guo *et al*. have reported that irradiation of the somatic pharynx results in a significant induction of bystander germ cell apoptosis^20^. Li Q *et al*. have performed local irradiation of either the posterior pharynx or the vulva of *C. elegans* as a comparison of the intra- and inter-system bystander effects, and investigated the spatial function of the oxidative DNA damage response by tissue-specific RNA interference^21^.

We report here the selective irradiation of 2-cell stage *C. elegans* embryos using the CPM at the AIFIRA facility (*Applications Interdisciplinaires de Faisceaux d’Ions en Région Aquitaine*)^22^. The aim of this study was to develop a methodology to micro-irradiate in a reproducible way the 2-cell stage *C. elegans* embryos with protons. Such a micro-irradiation of a dynamic 3D biological model with a fast cell division and rapidly evolving target volumes raises experimental challenges. The validation of this procedure includes an experimental validation of our ability to reproducibly induce radiation damage in embryos. Besides, Monte Carlo simulation on realistic 2-cell stage *C. elegans* phantoms was performed to characterize the specific energy absorbed in different biological compartments (chromatin, nucleus, cytoplasm and whole embryo), while considering different condensation states of chromatin throughout the cell division.

## Results

### Embryo phantom generation for monte carlo dosimetry simulation

**Figure 1:**
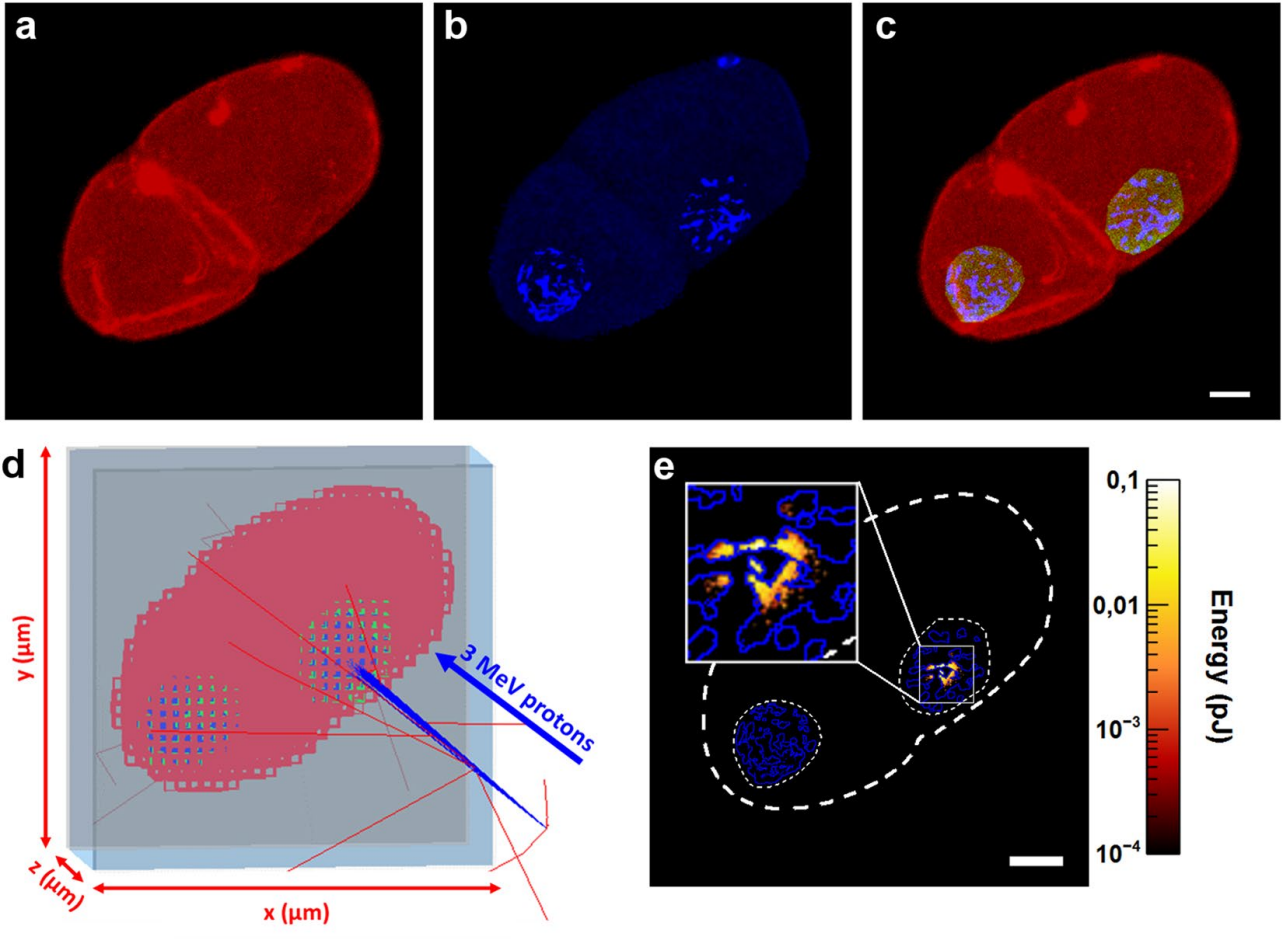
Monte-Carlo dosimetry and calculation of the energy deposit in 2-cell *stage C. elegans* embryo. **(a,b**) Confocal images of a 2-cell stage embryo stained with phalloidin (red) and Hoechst^33342^ (blue). **(c**) The chromatin (blue) and the embryo (red) are identified by applying an intensity threshold to separate fluorescent objects (chromatin) from the background. The nuclear volume (green) is outlined manually. (**d**) Voxelized geometry in Geant4 (low resolution) irradiated with 3 MeV protons. The incident protons are represented by blue lines, the red lines illustrate the trajectory of secondary electrons generated in air. **(e)** Z-projection of the energy deposit calculated using Geant4-DNA after irradiation with 10^3^ protons. The dashed outline corresponds to the contouring projection of the 3 volumes (chromatin in blue, nucleus and whole embryo in white). Scale bar: 5 µm.

When targeting sub-cellular structures with a microbeam, the radiation dose is delivered in micrometric areas. Standard dosimetry methods, based on the calculation of the absorbed dose at the macroscopic scale, are not fully relevant in this configuration. The spatial inhomogeneity of the irradiation leads to difficulties in defining the volume of interest, and the concept of absorbed dose is of limited use. In such situations, ICRU (International Commission on Radiation Units and Measurements) has introduced the concept of specific energy, defined as the ratio of the energy imparted to the mass of the volume of interest^23^. As the energy deposited by charged-particles depends significantly on the geometry of the target, we developed a three-dimensional realistic voxelized model of a 2-cell stage embryo. The energy deposits in 3 compartments were determined in our dosimetric study: (i) the chromatin, (ii) the nuclear volume, and (iii) the whole embryo. The geometries of these compartments were built from confocal microscopy acquisition of paraformaldehyde-fixed embryos. Phalloidin staining reveals the actin cytoskeleton of the 2-cell stage embryo in red (Fig. 1a) and Hoechst^33342^ staining revealed the chromatin in blue (Fig. 1b). As Phalloidin stains the actin-cytoskeleton which is absent in the nucleus, the nuclear volume is indirectly outlined by manual selection based on contrast rendering and cropping the area outside the region of interest (ROI). This ROI was then set as the green channel and the outside region was masked and left out from further processing (Fig. 1c). The resulting numeric phantom of a 2-cell stage was then implanted in Geant4 as a parametrized volume as shown in Fig. 1d. The microbeam spot size was set to 1.5 µm at the target (AB cell nucleus) position.

The methodology described previously by Barberet *et al*.^24^ was used to determine the energy imparted in each compartment as well as its distribution in space (Fig. 1e). The data obtained using this approach are summarized in Table 1. We calculated the corresponding specific energy as the ratio of the total energy deposit to the sum of the masses of voxels constituting the target (chromatin, nucleus or embryo). The mass of the different compartments was extrapolated by multiplying the sum of voxel volume constituting each compartment by liquid water density. We noted that the energy is deposited exclusively in the irradiated nucleus AB. The non-irradiated nucleus P1 does not receive any energy deposit (Fig. 1e). The statistical uncertainties related to the Monte-Carlo simulation are below 2%.

**Table 1:** Summary of the calculated and simulated data on realistic 3D-rendering of a 2-cell stage *C. elegans* embryo.

	Energy deposit per proton (fJ)	Mass (kg)	Specific energy per proton (mGy)
Chromatin	0.38 ± 0.01	2.2 × 10^−14^	18.2
Nuclear volume	9.47 ± 0.08	2.1 × 10^−13^	45.2
Whole embryo	29.77 ± 0.22	1.3 × 10^−11^	2.3

The data shown in the table are obtained from calculations in the *C. elegans* embryo in Fig. 1. The reported energy deposits are mean values ± standard deviations. These statistical uncertainties are related to the Monte Carlo calculations and they are below 2%.

**Figure 2:**
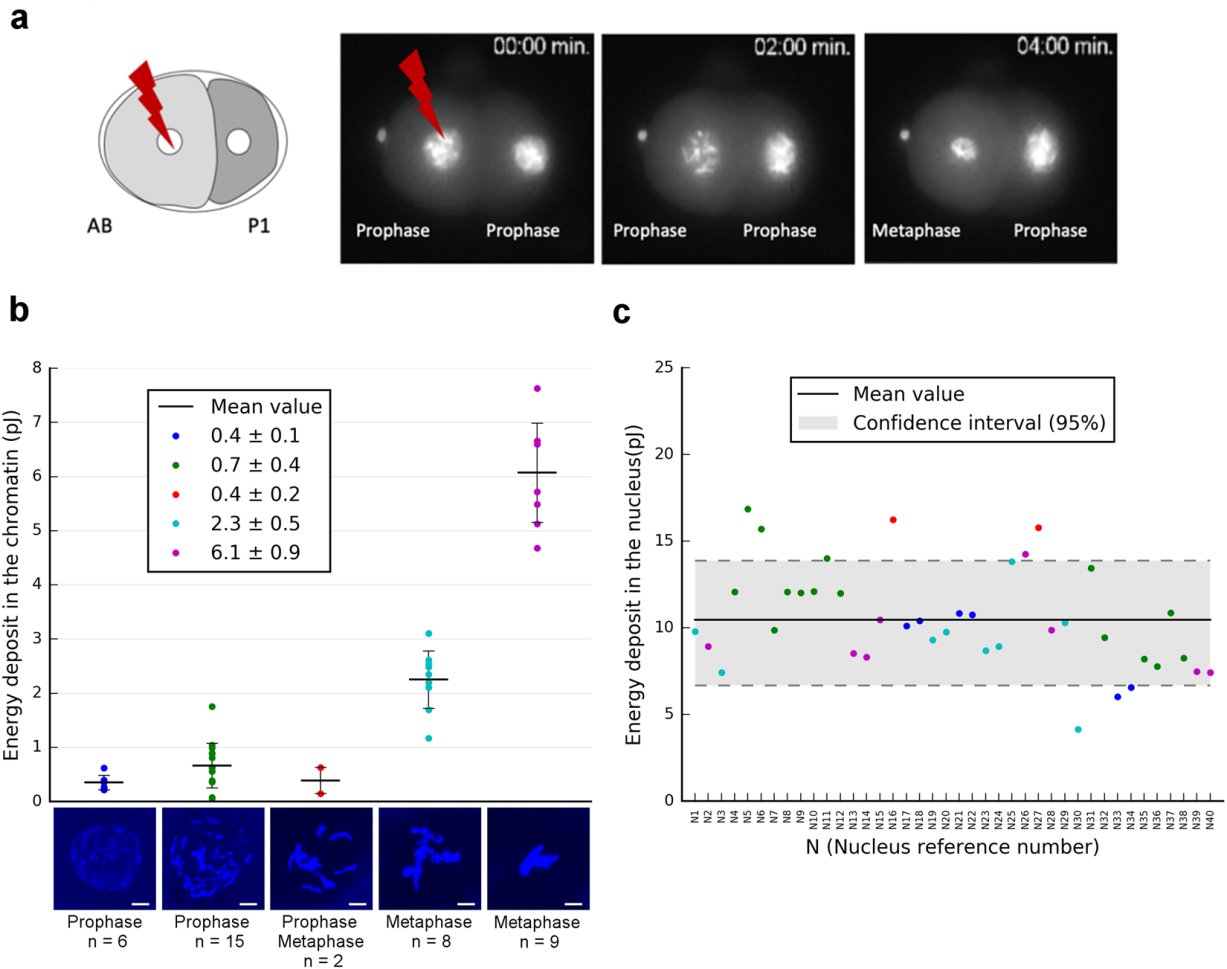
Monte-Carlo dosimetry and calculation of the energy deposit in the chromatin and nucleus. **(a**) First cell division of 2-cell stage embryos using strains expressing the histone H2B::GFP that revealed the chromatin condensation state at irradiation time. (**b**) Calculation of the total energy deposit (for 10^3^ protons) in the chromatin depending on its condensation status during the mitosis. 2D-projections of 5 different chromatin distributions revealed by Hoechst^33342^ (blue) and recorded in 40 embryonic cells by confocal microscopy. Scale bar: 3 µm. (**c**) Total energy deposit in the nucleus calculated using the same methodology described above for 40 embryonic cells (with 10^3^ protons). The scatter-plot point colours represent the corresponding chromatin condensation state colours from (**b**).

Since cell division is a rather fast process in *C. elegans* embryos as illustrated in Fig. 2a, we investigated the impact of the DNA/chromatin condensation level on the energy imparted to the chromatin (Fig. 2a). For this purpose, 40 single cells within 2-cell stage embryos were simulated after irradiations with 10^3^ 3 MeV protons. Based on the chromatin condensation status throughout the cell cycle progression of the AB cell (*t* = 0 *to 4* *min*), 5 distinct chromatin distributions could be discriminated from confocal imaging (Fig. 2b). They are representative of distinct mitosis progression states. The calculated energy imparted to the chromatin is different depending on the chromatin distribution observed in prophase or metaphase (Fig. 2b).

Nevertheless, as illustrated by these results, even if some variation of the chromatin distribution is found in prophase, it does not affect drastically the energy deposit in the chromatin at the irradiation time (0.5+/− 0.15 pJ). This point is crucial in the context of the dose-response assessment of biological radiation-induced effect. In addition, as illustrated in Fig. 2c, the calculated energy deposit in the 40 embryonic cell nuclei is around 10.5 +/− 2.8 pJ per nucleus (+/− 3.8 as 95% Confidence Interval (IC)). This 25% fluctuation is related to the variation of the nuclear volume from one embryo to the next. Indeed, the mean nuclear thickness traversed by the incoming protons was evaluated to 6 µm with a variation of 1.5 µm. Despite the additional uncertainties due to the manual segmentation of the nuclear volume, this is confirmed by the 2D fluorescence images of the GZ264 strain. We observed that the nucleus diameter can vary from 6.5 to 8.5 µm depending on the cell cycle stage. Assuming a spherical shape, this corresponds to a 25% variation of the nucleus thickness. Note that if we consider the total energy imparted to the nuclear volume, only 4% of this energy is absorbed by the chromatin in prophase (Table 1). This ratio can increase up to 60% in metaphase.

### Selective and targeted irradiation reveals HUS-1::GFP nuclear foci

**Figure 3:**
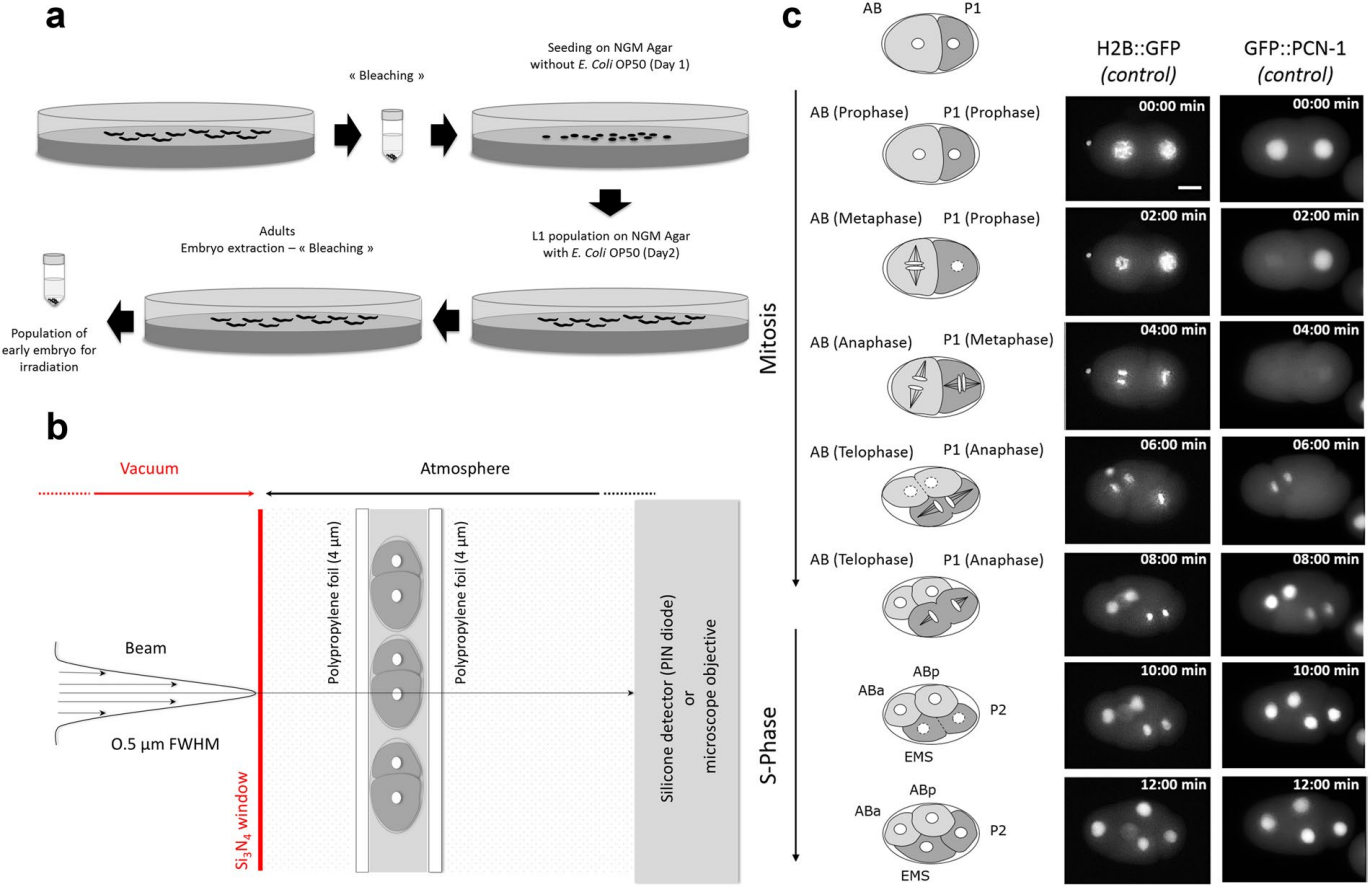
Schematic representation of the different steps needed for micro-irradiation of early *C. elegans* embryos in development. (**a**) Preparation of large populations of early *C. elegans* embryos by bleaching. (**b**) Scheme of the experimental end-station. The embryos are maintained between two thin polypropylene foils (4 µm in thickness) and the AB nucleus is targeted using online fluorescence microscopy. The beam is positioned on the targeted cell by means of electrostatic deflection and an exact number of protons is delivered and counted downstream the sample using a silicon detector. (**c**) *Timing of cell division in early C. elegans embryo* of MG152 (H2B::GFP) and GZ264 (GFP::PCN-1) strains. The nucleus is marked as a white circle in the middle of the cell. Asymmetric division of the P0 cell generates a larger, anterior AB cell and smaller, posterior P1 cell. Mitosis entry is indicated by nuclear breakdown (illustrated with dashed circle) and is accompanied by spindle rotation. Before cytokinesis of AB is completed, the posterior P1 cell enters mitosis (dashed circle). Cell divisions result in a four-cell embryo, with the daughter cells ABa and ABp derived from AB, and EMS and P2 derived from P1. Scale bar: 10 µm.

We adapted well-established CPM irradiation techniques previously developed for *in vitro* culture studies^2,4^. Embryos, prepared as described in Fig. 3a, were kept in M9 medium between two stretched polypropylene foils. The chosen foils are thin enough (4 µm) to allow the charged-particles to traverse through the sample and be detected downstream (Fig. 3b). This procedure resulted in the reproducible placement of the embryos in the focal plane of the objective lens. Under such conditions, we found that the slight compression of the polypropylene foils has no significant effect on the first cellular divisions. *C. elegans* embryos undergo fast, cleavage-type divisions in which the cell volume decreases after every cell division. The cell cycle in the early embryo stages is only composed of consecutive rounds of DNA synthesis (S phase) and cell division during mitosis (M phase), with no gap phase until the 28-cell stage^25,26^. The 2-cell stage embryos are formed of a larger anterior blastomere, AB, and a smaller posterior blastomere, P1, which have different fates and cell division timings; AB is divided 2 min before P1^21^. AB and P1 are oriented along the anteroposterior axis (Fig. 3c). In our experimental conditions, the first cellular divisions were found to be similar to the ones observed in conventional experimental conditions with *C. elegans* zygote exhibiting rotational holoblastic cleavage. We chose to irradiate selectively and specifically the nucleus of the AB cell. This corresponds to time 0 of our time lapse recording (*t* = *0* *min*).

**Figure 4:**
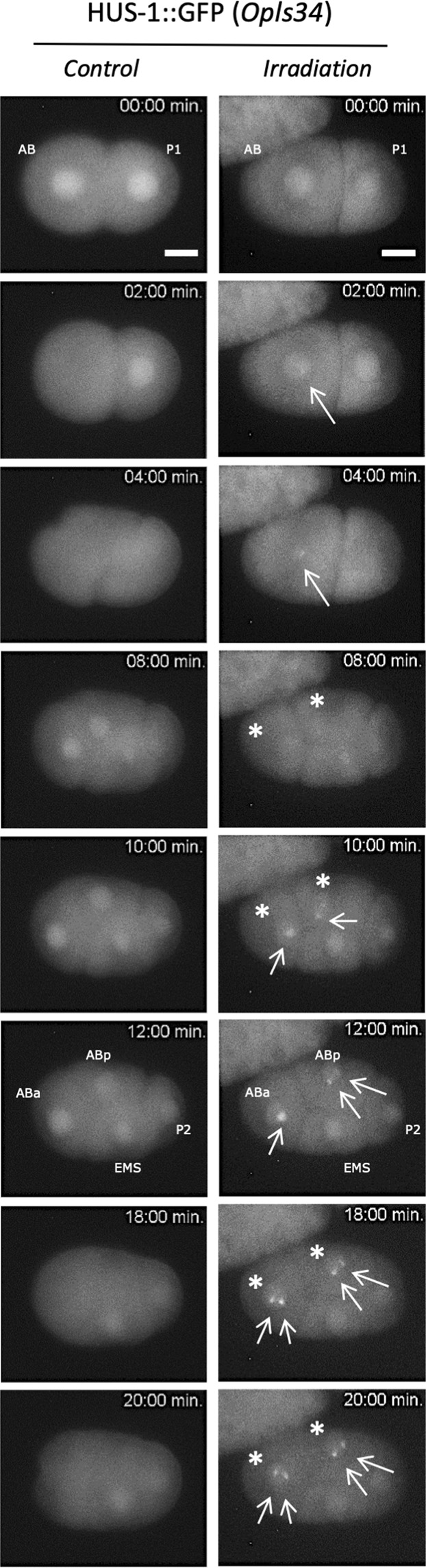
Real-time analysis of micro-irradiated AB nucleus revealed the subcellular relocalization of HUS-1::GFP (foci). First cell divisions of 2-cell stage embryos are observed using a strain expressing HUS-1::GFP (*opls34*). Before irradiation HUS-1::GFP is homogenously distributed in nuclei (*t* = *0* *min*). The AB cell nucleus is targeted with protons at t = 0 min. In micro-irradiated AB nucleus of HUS-1::GFP embryo, a focus, indicated with white arrow (→), appears just before the first cell division of AB (*t* = 2 min.) and reappears in the daughter cells ABa and ABp indicated with stars (*, right). We never observed foci in both non-irradiated embryo (control) and neighbouring non-irradiated nuclei (P1, EMS, and P2). Irradiated embryos (*opls34* = 10^4^ protons) were observed in real time following irradiation. Scale bar: 10 µm.

In order to obtain a direct visualization of the radiation-induced DNA damage in the *C. elegans* embryo, we used the WS1433 (*opIs34[hus-1::GFP])* strain. In *C. elegans*, HUS-1 is a conserved nuclear protein that is expressed in early embryos and the adult germline. In proliferating embryo nuclei, HUS-1::GFP expression is low, homogeneously distributed and limited to the nucleus as shown in Fig. 4 (*Control*). HUS-1 is part of the 9:1:1 complex belonging to DNA damage checkpoint protein acting as a DNA damage sensor. Thus, it is required for DNA damage-induced cell cycle arrest and apoptosis^27^ and is essential for genome stability, as demonstrated by an increased frequency of spontaneous mutations, chromosome non-disjunction, and telomere shortening^28^. When DNA damage occurs, HUS-1 is relocated into the nucleus in distinct foci that overlap chromatin. These foci are likely sites of double-strand breaks (DSBs). Following targeted micro-irradiation with 10^4^ protons, HUS-1::GFP relocated swiftly and formed a distinct and bright focus at the site of irradiation (Fig. 4, Supplementary Videos S1 and S2). However, we observed some variability between the nuclei in terms of diameter and number of foci per nucleus. HUS-1::GFP foci were maintained during the mitotic division and found in the daughter cell nuclei ABa and ABp. HUS-1::GFP foci were only detected for the highest irradiation specific energy (180 Gy/chromatin) in 8/9 embryos (additional data are shown in Fig. S2) harbouring nuclear HUS-1::GFP foci (total of 9 irradiated embryos).

By contrast, we never observed the occurrence of HUS-1::GFP foci in non-irradiated nuclei and their vicinity (P1 and related daughter cells), as well as in control embryos and in irradiated embryos with the lowest specific energy. In addition, no HUS-1::GFP foci were detected when the embryos were irradiated with 10^3^ protons (data not shown).

### Consequences of the micro-irradiation on chromosomes in 2-cell stage embryo

**Figure 5:**
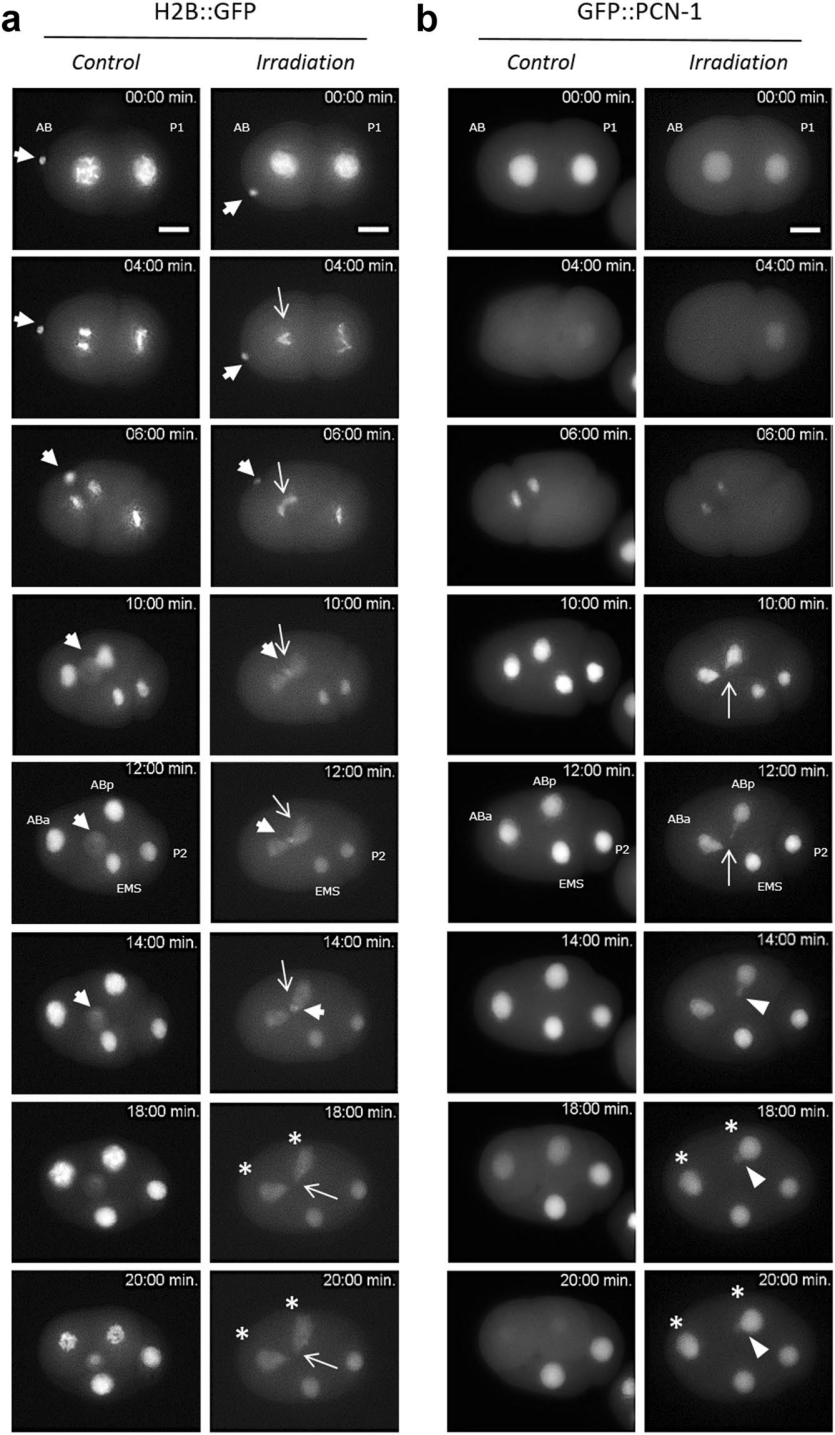
Time-lapse imaging of micro-irradiated AB nucleus revealed chromatin bridges and synchronization disruption of cell divisions within the 4-cell stage *C. elegans* embryos. First cell division of 2-cell stage embryos is observed using integrated strains expressing the histone H2B::GFP and GFP::PCN-1. Images are obtained at the same magnification. Scale bar 10 µm. The nucleus of the AB cell is targeted with 10^4^ protons at *t* = *0* *sec* (*Irradiation*). (**a**) H2B::GFP is bright and reveals the chromatin condensation during the different mitotic steps (*Control*). Note the presence of the polar bodies at the anterior part of the embryo (extranuclear H2B::GFP labelling, ➔). In micro-irradiated nucleus, H2B::GFP allows for the visualization of *in situ* and real time formation of DNA bridges (→) between the two dividing daughter cells ABa and ABp. Polar bodies are indicated with white arrows (➔) (**b**) In GZ264 embryos, the nuclear GFP::PCN-1 signals the S-phase and its loss suggests the nuclear membrane breakdown (mitosis). In the irradiated nucleus of GFP::PCN-1 embryo, the formation of DNA bridges during the first division can be clearly seen during the mitosis until their breakdown and the formation of extra-nuclear DNA (►). Disruption of the cell division synchronization within the 4-cell stage *C. elegans* embryo is also seen (*). The PCN-1::GFP signal helps to distinguish a clear shift of cellular division between irradiated and non-irradiated embryos (*). The formation of DNA bridges was never observed in non-irradiated nuclei (P1, EMS, P2) and or non-irradiated embryos.

The cellular consequences of targeted irradiation were followed using a strain (MG152) expressing the histone H2B::GFP fusion protein. This protein allows the imaging of both mitotic chromosomes and interphase chromatin in intact, living embryos. Embryos expressing H2B::GFP were observed using time-lapse imaging to determine the pattern of chromatin staining in interphase and mitosis after irradiation of the AB nucleus. As observed in control embryos, H2B::GFP enables highly sensitive chromatin detection in all phases of the mitosis. Figure 5a shows control embryos with chromosome condensation, the formation of a regular metaphase plate, a sudden and complete separation of anaphase chromosomes without the presence of chromatin bridges between daughter cells (ABa, ABp, EMS, and P2).

In contrast, in the irradiated embryos, abnormalities in chromosomal morphology became apparent during the first mitotic division of the AB cells, with chromosomes more poorly condensed than in non-irradiated embryos during anaphase (Fig. 5a, *irradiation*). In addition, H2B::GFP provided a remarkable level of sensitivity which allowed the detection of chromatin bridges. These are lagging strands of DNA between chromosomes that are separating during the mitotic phase and became obvious between the two daughter cells upon reaching the end of anaphase. These chromatin bridges remained until cytokinesis occurred, when the cell division broke them to produce a “cut” phenotype, resulting in the uneven distribution of genomic DNA to daughter cells. Chromatin bridges were clearly observed in all the embryos irradiated at the highest specific energy (5/5, additional data are shown in Fig. S3). The P1 blastomeric division, which occurs 2–3 minutes later, was normal; the chromosomes appeared well condensed in both controls and in irradiated embryos. No chromatin bridges were identified between the P1 daughter cells, and their nuclear morphology under epifluorescence was normal (Fig. 5, Supplementary Videos S3–S6).

Epifluorescence images of H2B::GFP embryos show the presence of one polar body at the anterior part of the embryo. Polar bodies are visible as small membrane-bound oval structures between the eggshell and the embryo, and are clearly visible with histone H2B::GFP in living embryos. The first polar body is 2N, born during eggshell secretion, and trapped between eggshell layers. The second polar body 1N is born after eggshell formation and is in contact with the embryo. At the 2-cell stage, the second polar body is on the surface of the anterior AB cell. During AB cell division, the second polar body is drawn between cells by the ingression furrow. At the 4-cell stage, the second polar body is inside a membrane within one of the AB daughter cells. In irradiated embryos, the presence of the chromatin bridges between the two daughter cells ABa and ABp affected and stopped the migration of one of the polar bodies until the cytokinesis occurred (Fig. 5a). Despite risking aneuploidy, *C. elegans* embryonic cells internalize the polar body and degrade polar body chromosomes inside a phagolysosome^29^. Here, we showed that the fate of the polar bodies seems to be modified in presence of the DNA bridges.

### Real-time analysis of micro-irradiated nuclei reveals synchronization disruption of cell divisions within the 4-cell stage *C. elegans* embryos

The dynamics of DNA replication in the *C. elegans* 2-cell stage embryo were estimated with a transgenic stain expressing PCN-1, the *C. elegans* orthologue of PCNA (proliferating cell nuclear antigen)^30^, fused to GFP. PCN-1 acts as a processivity factor for DNA replication and is one of the last components incorporated into the replisome upon replication initiation^31^. We used the GFP::PCN-1 chimera to visualize S-phase in early embryos and during subsequent cell or nuclear cycles. In other organisms, its accumulation in nuclear foci has been previously demonstrated to report on the active sites of DNA replication^32,33^. In time-lapse epifluorescence images of developing 2-cell stage embryos, GFP::PCN-1 is bright and reveals the AB and P1 nuclei. It is restricted to the nucleus when an entire nuclear membrane is formed. Nuclear membrane breakdown releases the GFP::PCN-1 in the cytoplasm (Fig. 5b, Supplementary Videos S5 and S6). The nucleus of AB cell was targeted with 10^4^ 3 MeV protons (t = 0 min). We did not observe GFP::PCN-1 foci but, similarly to what was observed in H2B::GFP strain, we noted the formation of chromatin bridges during the first division (Fig. 5b, t = 10 min., additional data are shown in Fig. S4). The formation of such chromosomal abnormalities was never observed in non-irradiated nuclei (Fig. 5b, EMS and P2 cells, n = 9) or in non-irradiated embryos (Fig. 5b, control, n = 5). The cytokinesis induced the chromosomal bridge breakage and the formation of an extra nuclear DNA fragment (suggesting micronucleus formation) excluded from the nucleus. The formation of nuclei around mis-segregated chromosomes and chromosomal fragments apparently contributes to the formation of micronuclei, some of which with very little DNA (Fig. 5b).

The disruption of the cell division synchronization within the 4-cell stage *C. elegans* embryo is shown in Fig. 5b and Supplementary Fig. S1 (*t* = 30 *min*). In control embryos, the loss of the nuclear localization of GFP::PCN-1 is observed in ABa and ABp cells at *t* = 20 *min*. In irradiated embryos, the GFP::PCN-1 signal is still present at this time stamp and restricted to the nucleus in these cells. This is also underlined by comparing the time of division between ABa, ABp and EMS, P2. EMS, P2 entered mitosis before ABa and ABp in irradiated embryos. This is never observed in controls. By extending the time-lapse imaging until the 8-cell stage, we could observe that chromosomal bridges appear during the mitotic division of ABa and ABp as well, suggesting the maintenance of a genetic instability (Supplementary Fig. S1).

## Discussion

The aim of targeted irradiation is to induce damage localized in specific cellular compartments. Laser micro irradiation is the most commonly used method to achieve this goal mainly because one can use the same microscope to perform irradiation and observation. In addition, the easy access to different wavelengths for irradiation allows the induction of specific cellular damage like SSB, DSB or base lesions with a varying range of doses^34^. On the contrary, targeted irradiation using a CPM requires the use of distinct equipment for observation and irradiation, thus increasing the number of key parameters to control such as alignment of irradiation beam with the microscope, specific sample preparation, time-lapse imaging and irradiation synchronization. Despite this complexity, CPM offers a unique capability of irradiation dose control, down to a single particle^22,35^. In addition, with direct access to charged particle beams instead of photons, CPM plays a major role in the study of ionizing radiation in biology. In the present study, we report the possibility to extend this approach to specific cells in a developing multi-cellular organism.

Single cell nuclei can be targeted with a controlled number of charged-particles in living 2-cell stage *C. elegans* embryos and followed-up by time-lapse imaging. We could visualize radiation-induced DNA damage, and undoubtedly DSBs, a few minutes after irradiation in live and intact early *C. elegans* embryos with 10^4^ protons (equivalent to 180 Gy in the chromatin). Likewise, HUS-1::GFP radiation-induced foci were also observed in the daughter cells, indicating a persistence of altered DNA through the division cycle in agreement with the absence of active cell cycle checkpoints during DNA damage response in the early *C. elegans* embryo^36^. The production and maintenance of DNA bridges through the successive cell divisions have been shown in several *C. elegans* strains. We also observed that these DNA bridges alter the cell division timing, inducing a cell cycle asynchrony and modifying the movement of the cells within the irradiated embryos (Fig. 5b and supplementary Fig. S1). Our experimental approach is complementary to the well-established laser micro-irradiation^34,37^ and can complement it by producing more representative damage of ionizing radiation effects. CPM irradiation, commonly used on adherent mammalian cells, can be adapted to target specific cells in a developing multi-cellular organism to address the question of the *in vivo* radiation-induced consequence on a specific cell lineage, the cell-to-cell communication, and the low-dose effect. Data from the voxelized model indicate that the generation of DNA bridges and genomic instability observed experimentally require an energy deposit in the chromatin above 4 pJ (corresponding to a specific energy of about 180 Gy). This could be due to the limited sensitivity of our fluorescent markers or to the lack of specificity for the detection of the radiation-induced damage. But it could also suggest the existence of a dose-threshold below which no sufficient radiation-induced effect would be produced on genomic DNA during the first cell division of AB.

The simulation and mapping of the energy deposited in three dimensional phantoms allows us to correlate the energy deposited in each comportment (chromatin and nucleus) to the radiation-induced damage. First, we clearly observed that no energy deposit is found in the non-targeted nucleus and cell (P1). Second, Monte-Carlo simulations indicate that the biological effects described here (DNA damage foci, DNA bridges and cell cycle delay) appear at rather high specific energies, *i.e*. 180 Gy in the chromatin of the targeted nucleus (AB). Third, the energy deposits at various stages of the cell division show a variation of 25% correlated to the variation of the nuclear volume. Fourth, the fraction of energy imparted to the chromatin shows large fluctuations considering various phases of the division, ranging from only 4% in prophase to about 60% in metaphase. Fifth, as the specific energy imparted to the chromatin depends mainly on its condensation state, we verified that only limited variations in terms of energy imparted to the chromatin are expected between samples at the time of irradiation. Indeed, even if the precision in time at which a cell nucleus can be targeted is limited by the visual recognition of specific cell division patterns, a delay in irradiation timing below 2 minutes (chromatin in prophase, Fig. 2b) has no significant influence on the specific energy deposited in the chromatin.

The ability to assess energy deposits and specific energy in various cell compartments relies on the possibility to handle parametrized volumes obtained from fluorescence microscopy in the Geant4 simulation toolkit at the sub-micrometre scale. We believe that new geometrical models developed in the frame of the Geant4-DNA extension, will strengthen biological damage prediction at the DNA molecule scale in cell-based phantoms^38^, particularly by including the production of DNA single and double strand breaks induced by the direct and indirect effects of oxidative radical species on the cell DNA content^39^.

## Materials and Methods

### Worm strains and culture

*C. elegans* worm strains were maintained on nematode growth medium (NGM) agar plates and fed *ad libitum* with *Escherichia coli* strain OP50 at 20 °C, according to standard protocols (Brenner, 1974). We used the following transgenic *C. elegans* strains carrying appropriate fluorescent markers: MG152 (*xsIs3[HisH2B::GFP; rol-6(su1*00*6)]*, WS1433 (*opIs34[hus-1::GFP])*, GZ264 *(isIs17[pGZ265:pie-1::GFP-pcn-1(W0D2.4)]), SA250* (tjIs54 [pie-1p::GFP::tbb-2 + pie-1p::2xmCherry::tbg-1 + unc-119(+)]. tjIs57 [pie-1p::mCherry::his-48 + unc-119(+)]). The Caenorhabditis Genetics Centre (CGC, University of Minnesota) provided these *C. elegans* strains and the *E. Coli* OP50.

### Preparation of large population *C. elegans* embryos

Embryos were isolated from synchronized populations of young gravid hermaphrodites, treated with hypochlorite solution (“Bleaching”) (Fig. 3a). The bleaching technique was used for synchronizing *C. elegans* cultures at the first larval stage (L1). The principle of the method lies in the fact that worms are sensitive to bleach while the egg shell protects the embryos from it. After treatment with an alkaline hypochlorite solution and rinsing, embryos were maintained on NGM agar plates without food, which allows hatching but prevents further development. Once hatched, the L1 larvae were transferred onto NGM agar plates seeded with OP50 *E. coli* as a food source until the worms reached the adult stage. A second and final bleaching step just before irradiation was performed. These synchronized populations of young gravid hermaphrodites from standard, well-fed culture stocks were collected with M9 buffer (3 g/l KH_2_PO_4_, 6 g/l Na_2_HPO_4_, 5 g/l NaCl, 1 mM MgSO_4_) and washed three times with sterile water to remove bacteria. Then, worms pelleted *via* centrifugation (2 min., 2000 rpms, room temperature) were treated with a freshly prepared alkaline hypochlorite solution (1.5% (v/v) NaOCl, 1 M NaOH). The suspension was swirled every 2 minutes with vortex-mixing (~6 min.). The released embryos were pelleted *via* centrifugation at 2000 rpms for 2 minutes. Supernatant was carefully discarded and embryos washed three times with M9 buffer followed by centrifugation at 2000 rpms for 2 min. The pelleted embryos were suspended in 50 µL of fresh M9 and an aliquot ~2 µl was directly positioned and used in experimental conditions for micro-irradiation and live imaging using a custom-made support dish described previously by Muggiolu *et al*.^35^. This sample holder provides a stable long-term environment for microscopic analysis and micro-irradiation experiments. The elapse time between “bleaching” and irradiation was shortened in order to favour the presence of both one-cell and 2-cell stages embryos.

### Micro-irradiation

3 MeV protons (H^+^, LET = 12 keV.µm^−1^ in liquid water) were accelerated by a 3.5 MV electrostatic accelerator (Singletron, High Voltage Engineering Europa, The Netherlands) present in the AIFIRA facility^40^. In order to target a single cell of a 2-cell stage embryo, the beam was strongly collimated to reduce the particle flux to a few thousand ions per second on target and focused using a triplet of magnetic quadrupoles to achieve a sub-micron resolution under vacuum. After extraction in air, the beam spot size is 1.5 µm. The delivered dose was controlled by counting the particles with a PIN diode installed downstream the sample on the microscope objective wheel. Embryos were irradiated with two doses: 10^3^ and 10^4^ protons per AB cell nucleus.

### Imaging

Embryos were positioned for micro-irradiation and live imaging using a custom-made support dish described by Muggiolu *et al*.^35^. A drop of 3-µL of M9 medium containing a suspension of freshly extracted embryos was deposited and maintained between two 4-µm thick polypropylene foils (Goodfellow) stretched on a rigid frame made in PEEK (Polyether ether ketone) by means of a clip, thus avoiding the use of any glue. These two foils were slightly stretched to close the dish and maintained the *C. elegans* embryos within a thin layer of M9 which still allowed the traversal of the incoming protons. The M9 medium was kept at +19 °C and the mounted sample kept at 20 °C (+/− 2 °C). All along the experiments, the temperature was monitored and recorded using a thermal probe (PicoLog TC-08). AB nuclei were targeted in their centre based on fluorescence of either GFP tagged HUS-1, histone H2B, or PCN-1. The AB cell was identified due to its larger volume than P1 cell. Embryos were imaged using an inverted fluorescence microscope (AxioObserver Z1, Carl Zeiss Micro-Imaging GmbH) and an x63 objective (LD Plan-Neofluar 63x/0.75) positioned horizontally at the end of the beamline. Images were captured at 10 sec periods using an AxioCam™ CCD camera and directly transferred to a personal computer through a Firewire 400 connection. The irradiation was triggered when the nucleus reached a central position in the AB cell. Time series of micro-irradiated embryos were created using *Image J* software (http://rsbweb.nih.gov/ij/). A minimum of five embryos were analysed for each experimental group.

### Immunofluorescence staining

Freshly extracted embryo populations were immediately fixed in cold 4% (w/v) paraformaldehyde and their eggshells were freeze-cracked by placing at −20 °C during 15 min. Then, embryos were pelleted *via* centrifugation (2 min., 2000 rpms, RT) and paraformaldehyde replaced by cold acetone for permeabilization (2 min, −20 °C) and, finally twice washed in M9. After fixation and washing, M9 was removed and replaced by a freshly prepared solution of phalloidin-AF^594^ (10:1000 (v/v), Molecular Probes) and Hoecsht^33342^ (2:5000 (v/v), Molecular Probes). Embryos were incubated overnight at RT under gentle agitation and then washed *via* two series of centrifugation (2 min. 2000 rpms) with M9. The supernatant was discarded and the pelleted embryos suspended in 2–3 drops of Prolong Gold Antifade reagent (Molecular Probes) and transferred by pipetting for mounting between glass slides using ProLong™ Antifade Gold Reagent (Invitrogen). Three-dimensional images were acquired with a Leica DMRE TCS SP2 AOBS confocal microscope (oil-immersion objective × 63, 1.4 NA), assembled, and reconstructed using *Image J* software.

### Monte Carlo simulation

Geant4-DNA^41–44^, the open source very low energy extension of the Geant4 Monte Carlo simulation toolkit^45–47^, was used for this work (release Geant4.10.3.p01). We used a physics list based on the recommended “G4EmDNAPhysics_option4” constructor^44^. The Geant4-DNA processes are all step by step processes; as such, they simulate explicitly all the physical interactions of ionizing particles in the irradiated medium and do not use any production cut-off. In the MeV range, the dominant physical processes affecting protons are nuclear scattering, electronic excitation, ionization, and charge exchange. Further details on the physical process classes can be found in the Geant4 documentation (http://geant4-dna.org/)^48^.

The primary beam was modelled as mono-energetic 3 MeV proton beam having a 1.5 µm FWHM Gaussian distribution. The 2-cell stage embryo of *C. elegans* was modelled as a parametrized geometry designated as “phantom” in the following. The phantom was simulated in the form of a voxel arrangement determined from images acquired with a confocal microscope. The methodology for converting confocal image data into a 3D phantom has been described previously in Barberet *et al*.^22^. The set of images of the 2-cell stage embryos acquired from the confocal microscope was transferred into the public domain ImageJ software (http://rsbweb.nih.gov/ij/) for 3D reconstruction (Fig. 1a,b). An intensity threshold was then applied for each colour channel to separate fluorescent objects from the background in each slice of the stack. The nuclear volume was defined as the green channel by manually selecting a ROI around the chromatin on the red channel based on contrast selection, and cropping the region outside the ROI (Fig. 1c). The chromatin, nuclear volume and embryo volume could be extracted into an individual text file containing: (i) the total number of voxels for the 3 channels (Red, Green and Blue), (ii) the voxel size along the 3 dimensions, (iii) a position shift in order to centre the embryo in the simulated irradiation dish, (iv) the list of each voxel’s position and material composition, as well as the intensity fluorescence content of the voxel.

The phantom could then be imported directly into the Geant4 simulation using the method described by Incerti *et al*.^11^. The implemented phantom was formed of multiple identical parallelepiped voxels having the same size, indicated in the form of the confocal images.

The simulations were performed for 10^3^ and 10^4^ incoming protons to reproduce the experimental conditions.

## Supplementary information

Supplementary Video S1: Time-lapse recording of control WS1433 strain 2-cell stage C. elegans embryo.

Supplementary Video S2: Time-lapse recording of micro-irradiated AB nucleus of WS1433 strain 2-cell stage C. elegans embryo revealed relocalisation of HUS-1::GFP (foci).

Supplementary Video S3: Time-lapse recording of control MG152 strain 2-cell stage C. elegans embryo.

Supplementary Video S4: Time-lapse recording of micro-irradiated AB nucleus of MG152 strain 2-cell stage C. elegans embryo revealed chromatin bridges.

Supplementary Video S5: Time-lapse recording of control GZ264 strain 2-cell stage C. elegans embryo.

Supplementary Video S6: Time-lapse recording of micro-irradiated AB nucleus of GZ264 strain 2-cell stage C. elegans embryo revealed chromatin bridges and synchronization disruption of cell divisions.

Supplementary Data 1
