# Supplementary material for: Monte-Carlo dosimetry and real-time imaging of targeted irradiation consequences in 2-cell stage Caenorhabditis elegans embryo
Source: Sci Rep. 2019 Jul 22;9:10568. doi: 10.1038/s41598-019-47122-7 (PMC6646656; doi:10.1038/s41598-019-47122-7)
Supplement: Supplementary file 7 — Supplementary Data 1 [file 41598_2019_47122_MOESM7_ESM.docx]

**Monte-Carlo dosimetry and real-time imaging of targeted irradiation consequences in 2-cell stage *Caenorhabditis elegans* embryo**

Eva Torfeh^(1,2)^, Marina Simon^(1,2)^, Giovanna Muggiolu^(1,2)^, Guillaume Devès^(1,2)^, François Vianna ^(1,2,#)^, Stéphane Bourret ^(1,2)^, Sébastien Incerti^(1,2)^, Philippe Barberet^(1,2)^ and Hervé Seznec^(1,2)*^

*(*1*) Université de Bordeaux, Centre d’Etudes Nucléaires Bordeaux Gradignan (CENBG), Chemin du Solarium, 33175 Gradignan, France*

*(2) CNRS, UMR5797, Centre d’Etudes Nucléaires Bordeaux Gradignan (CENBG), Chemin du Solarium, 33175 Gradignan, France*

Correspondence and requests for materials should be addressed to
P.B. (email: [barberet@cenbg.in2p3.fr](mailto:barberet@cenbg.in2p3.fr)) and H.S. (email: [herve.seznec@cenbg.in2p3.fr](mailto:herve.seznec@cenbg.in2p3.fr))

# Present address, François Vianna: *Institut de Radioprotection et de Sûreté Nucléaire, Bat.159, BP3, 13115 St-Paul-Lez-Durance, Cedex, France*

**Supplementary Data 1**

**
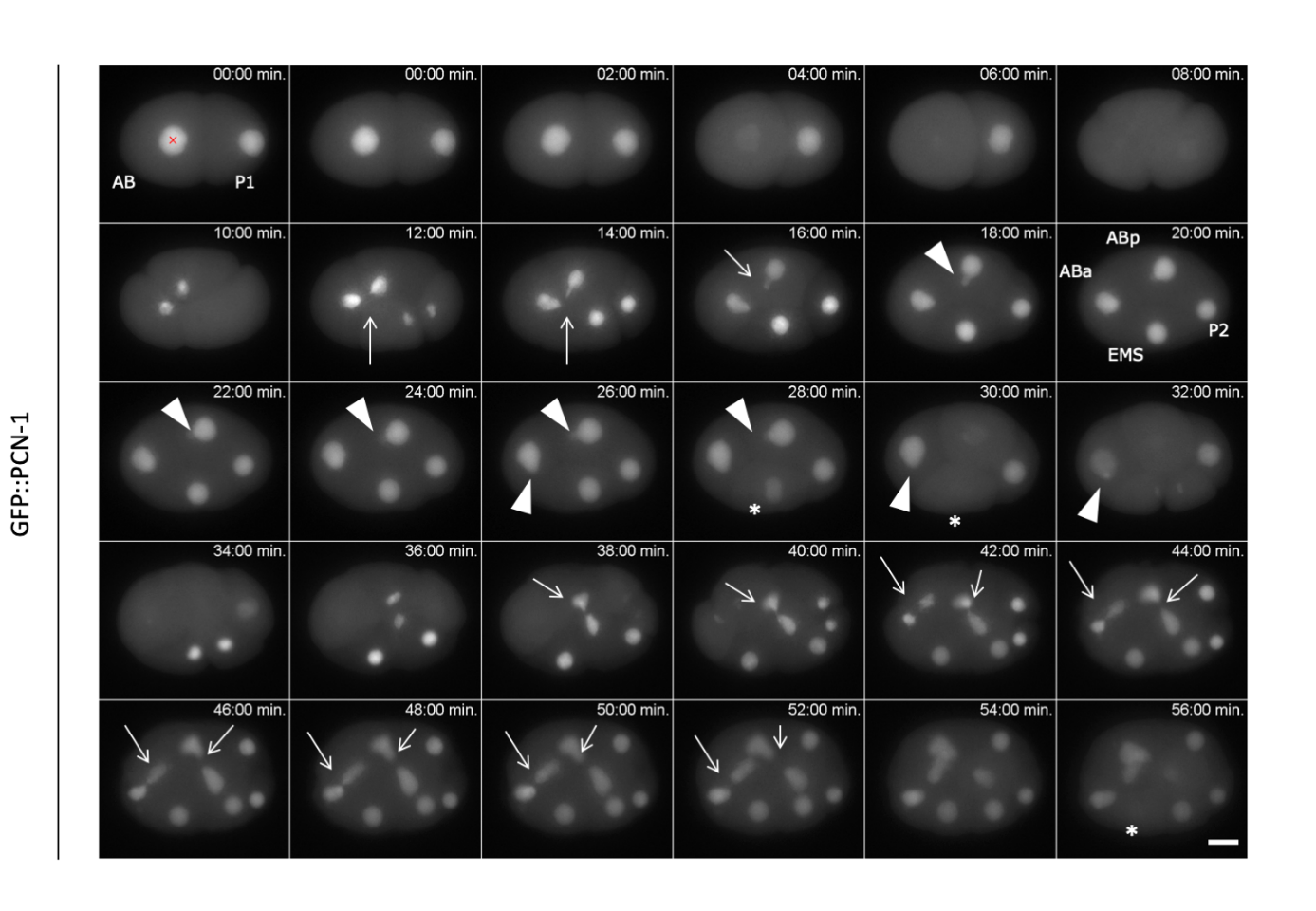
**

**Figure S1: Real-time analysis of micro-irradiated nucleus revealed genetic instability and synchronization disruption of cell divisions within the 8-cell stage *C. elegans* embryos.** Time-lapse epifluorescence images of developing 2-cell stage embryos expressing GFP::PCN-1 is bright and revealed the nucleus through cell division until the 8-cell stage. The nucleus of AB cell is targeted with 10^4^ protons at t=0 min. In micro-irradiated AB nucleus, the formation of DNA bridges (🡒) during the first division can be clearly seen during the mitosis until their breakdown and the formation of extra-nuclear DNA (▶). These alterations are maintained through the successive divisions. The disruption of the cell division synchronization within the 4-cell and 8-cell stage *C. elegans* embryo is also seen (*). The PCN-1::GFP signal helps to distinguish a clear shift of cellular division between irradiated and non-irradiated embryos (*). For example, EMS is dividing earlier than ABp (nuclear breakdown observed at *t=28 min*.). The formation of DNA bridges was never observed in non-irradiated nucleus (P1, EMS, P2) and or non-irradiated embryos. Scale bar: 10 µm.


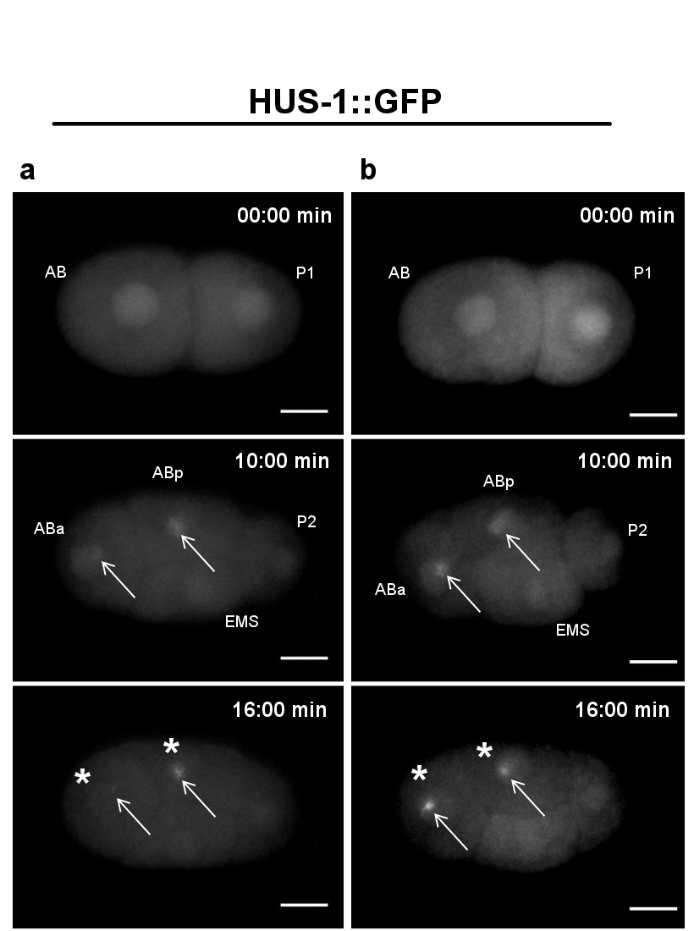


**Figure S2: Real-time analysis of micro-irradiated AB nuclei revealed the subcellular relocalization of HUS-1::GFP (foci).** First cell divisions of 2 different embryos (a) and (b) in 2-cell stage observed using a strain expressing HUS-1::GFP. Before irradiation HUS-1::GFP is homogenously distributed in nuclei (*t=0 min*). The AB cell nucleus is targeted with 10^4^ protons at *t=0 min*. In the daughter cells (ABa and ABp) of the micro-irradiated AB nuclei, foci indicated with white arrow (🡒), appear while we never observed foci in neighbouring non-irradiated nuclei (P1, EMS, and P2). Scale bar: 10 µm.

**
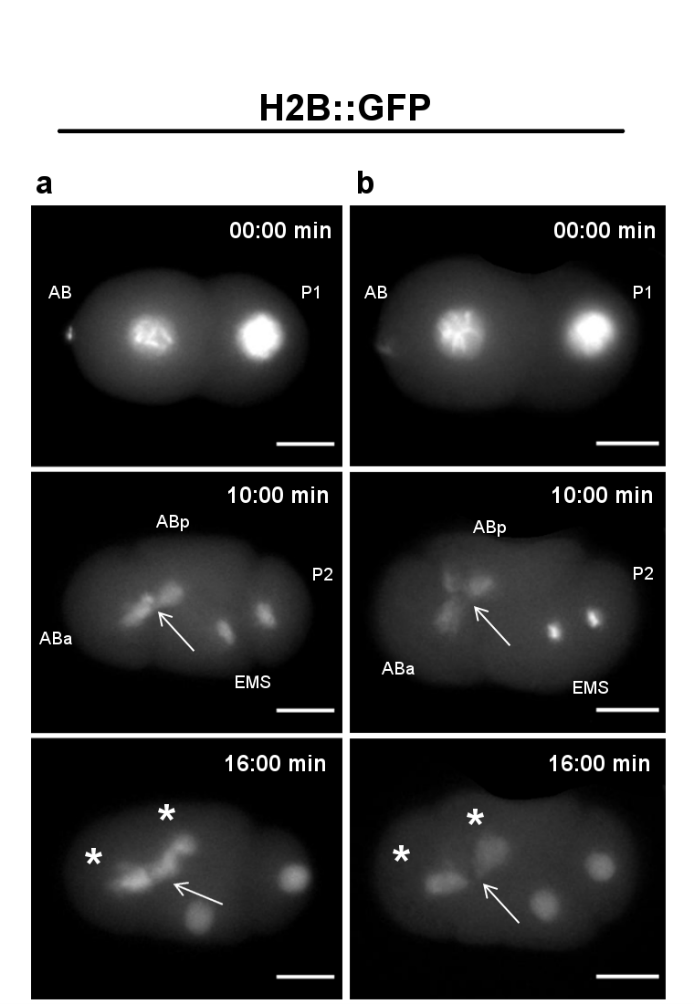
**

**Figure S3: Real-time analysis of micro-irradiated AB nuclei revealed chromatin bridges of cell divisions within the 4-cell stage *C. elegans* embryos expressing the histone H2B::GFP.** First cell divisions of 2 different embryos (a) and (b) in 2-cell stage observed using a strain expressing H2B::GFP. The nuclei of the AB cells were targeted with 10^4^ protons at *t=0 min.* In micro-irradiated nuclei, H2B::GFP allow for the visualization of *in situ* and real time formation of DNA bridges (🡒) between the two dividing daughter cells ABa and ABp. The formation of DNA bridges was never observed in non-irradiated nuclei (P1, EMS, P2). Scale bar 10 µm.


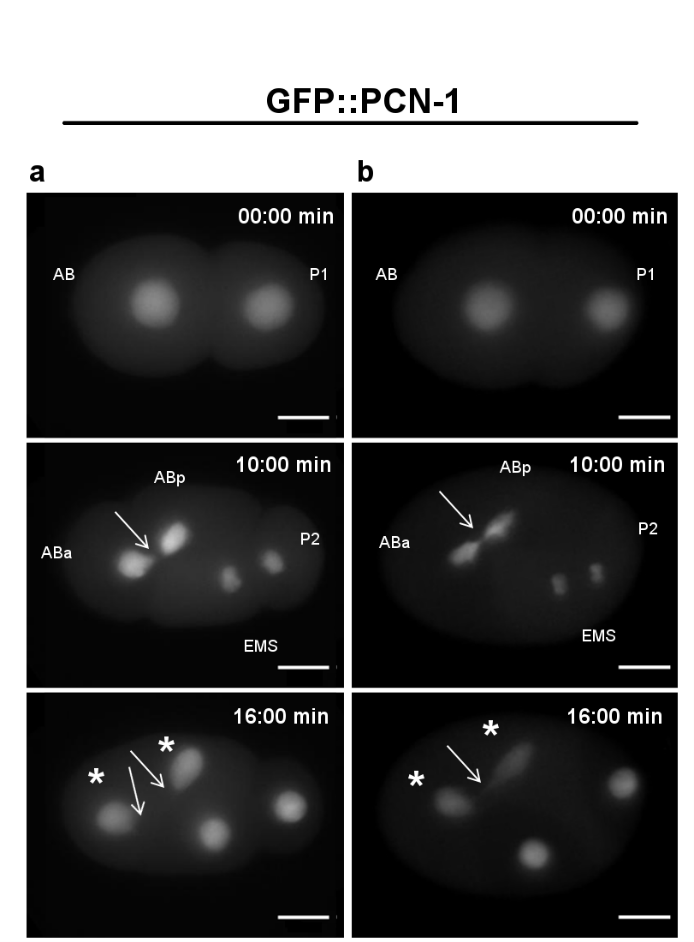


**Figure S4: Real-time analysis of micro-irradiated AB nuclei revealed chromatin bridges of cell divisions within the 4-cell stage *C. elegans* embryos expressing GFP::PCN-1.** First cell divisions of 2 different embryos (a) and (b) in 2-cell stage observed using a strain expressing GFP::PCN-1. The nuclei of the AB cells were targeted with 10^4^ protons at *t=0 min.* In micro-irradiated nuclei, the formation of DNA bridges during the first division can be clearly seen during the mitosis until their breakdown (🡒).The formation of DNA bridges was never observed in non-irradiated nuclei (P1, EMS, P2). Scale bar 10 µm.

**Supplementary Video S1: Time-lapse recording of control WS1433 strain 2-cell stage *C. elegans* embryo.** First cell divisions of 2-cell stage embryos expressing HUS-1::GFP distributed homogeneously in the nuclei during the cell division from 2-cell stage to 8-cell stage. Scale bar = 10 µm.

**Supplementary Video S2: Time-lapse recording of micro-irradiated AB nucleus of WS1433 strain 2-cell stage *C. elegans* embryo revealed relocalisation of HUS-1::GFP (foci).** The AB cell nucleus is targeted with 10^4^ protons at t=0 min. In the micro-irradiated AB nucleus of HUS-1::GFP embryo, a focus appears just before the first cell division of AB (*t*=2 min.) and reappears in the daughter cells ABa and ABp. Foci were not observed in non-irradiated nuclei (P1, EMS, and P2). Scale bar = 10 µm.

**Supplementary Video S3: Time-lapse recording of control MG152 strain 2-cell stage *C. elegans* embryo.** First cell divisions of 2-cell stage embryos expressing the histone H2B::GFP. H2B::GFP is bright and reveals the chromatin condensation during the different mitotic steps. Note the presence of the polar bodies at the anterior part of the embryo. Scale bar = 10 µm.

**Supplementary Video S4: Time-lapse recording of micro-irradiated AB nucleus of MG152 strain 2-cell stage *C. elegans* embryo revealed chromatin bridges.** The AB cell nucleus is targeted with 10^4^ protons at t=0 min. In micro-irradiated nucleus, H2B::GFP allows visualising of *in situ* and real time formation of DNA bridges between the two dividing daughter cells ABa and Abp. The formation of DNA bridges was never observed in non-irradiated nuclei (P1, EMS, and P2). Scale bar = 10 µm.

**Supplementary Video S5: Time-lapse recording of control GZ264 strain 2-cell stage *C. elegans* embryo.** First cell divisions of 2-cell stage embryos expressing GFP::PCN-1 that signals the S-phase and its loss suggests the nuclear membrane breakdown (mitosis). Scale bar = 10 µm.

**Supplementary Video S6: Time-lapse recording of micro-irradiated AB nucleus of GZ264 strain 2-cell stage *C. elegans* embryo revealed chromatin bridges and synchronization disruption of cell divisions within the 4-cell stage.** The AB cell nucleus is targeted with 10^4^ protons at t=0 min. In the irradiated nucleus of GFP::PCN-1 embryo, the formation of DNA bridges during the first division can be clearly seen during the mitosis until their breakdown and the formation of extra-nuclear DNA. Disruption of the cell division synchronization within the 4-cell stage *C. elegans* embryo is also seen**.** The formation of DNA bridges was never observed in non-irradiated nuclei (P1, EMS, and P2). Scale bar = 10 µm.
